# The effects of oral health education regarding periodontal health on non-dental undergraduates in southwestern China—exploring the feasibility of an e-learning course for oral health promotion

**DOI:** 10.1186/s12903-021-01476-5

**Published:** 2021-03-16

**Authors:** Zhiwu Wu, Mingming Li, Fangzhi Zhu, Lei Lei, Ran Cheng, Tao Hu

**Affiliations:** Department of Preventive Dentistry, State Key Laboratory of Oral Diseases, West China Hospital of Stomatology, Sichuan University, Chengdu, Sichuan China

**Keywords:** Periodontal diseases, Traditional course, E-learning course, Periodontal health education, Undergraduates

## Abstract

**Background:**

The high prevalence of periodontal diseases is an important oral health problem worldwide. It is necessary to increase public knowledge on and influence attitudes towards periodontal diseases in order to prevent them. However, the effect of oral health education (OHE) as a primary preventive method in China is unsatisfactory. The aim of this study is to investigate the feasibility of extending an e-learning course regarding periodontal health by comparing the effects of oral health education regarding periodontal health (OHE-PH) on dental and non-dental students and the effects between a traditional course and an e-learning course among non-dental students at Sichuan University.

**Methods:**

A quasi-experimental study with a pre-test and a post-test was performed. A total of 217 dental students and 134 non-dental students attended a traditional course; 69 non-dental students attended an e-learning course. Before- and after-course questionnaires about periodontal health knowledge, attitudes and behaviours were administered.

**Results:**

After the traditional/e-learning course, the knowledge of both dental and non-dental students about periodontal diseases and self-reported behaviours for gingival bleeding and oral care improved. The non-dental students reached or surpassed the level of dental students before the course. The non-dental students taking the e-learning course performed better in some areas than those taking the traditional course.

**Conclusions:**

OHE-PH was effective for dental and non-dental students. The e-learning course on OHE-PH was sufficient for improving knowledge and self-reported behaviours among non-dental undergraduates and was even better than the traditional course in some areas. The e-learning course may be an effective method for periodontal health education and oral health promotion among undergraduates.

## Background

Periodontal diseases are infectious diseases that affect tooth-supporting tissue and are among the most prevalent chronic diseases; they are a public health problem [1, 2]. The United States National Health and Nutrition Examination Survey showed that periodontitis had prevalence rates of 43.6% and 42% in 1999–2004 [3] and in 2009–2014 [4], respectively. The fourth National Oral Health Survey conducted in 2015 in China showed that 87.4% of adults between 35 and 44 years of age suffered from gingival bleeding [5], a sign of periodontal diseases [6, 7]. Oral health status, especially periodontal health status, was poor among Chinese populations [5]. Periodontal diseases are associated with systemic diseases, such as diabetes mellitus, adverse pregnancy outcomes, and cardiovascular diseases [8], which impose a major burden on society [9]. Fortunately, periodontal diseases can be prevented through interventions such as oral health education (OHE).

OHE is effective in improving knowledge and attitudes regarding oral diseases [10–14], thus contributing to disease prevention [15]. However, periodontal health knowledge and positive attitudes were unsatisfactory among Chinese adults [16, 17] despite the development of OHE during the past 30 years [16], for which there are many possible reasons. In China, OHE is mainly provided by dentists or specialists in Preventive Dentistry. In addition to OHE provided by dentists for patients in clinics, specialists in Preventive Dentistry conduct OHE in kindergarten, primary school, residential communities and nursing homes. Thus, schoolchildren [18] and elderly individuals are the most concerned populations in OHE. Young and middle-aged people, the workers of society, are busy with work and have limited time and opportunity to engage in OHE. OHE should be provided for more people, including adults, especially undergraduates. It has been reported that non-dental undergraduates had poor oral health knowledge [17, 19], and they will someday be parents and thus responsible for providing oral health instructions to their children. Furthermore, it is relatively feasible to provide OHE for university students through the established curriculum structure. Therefore, it is both necessary and possible to provide effective OHE for non-dental students, the benefits of which are cumulative.

In addition to considering the different target populations, the content of OHE should also be adapted. The concept of OHE should be different in accordance with the various targeted populations in kindergarten, primary/middle/high schools, universities, dental schools, workplaces and elderly communities. For schoolchildren and elderly individuals, OHE is simplified and designed to be vivid to ease their understanding. However, simplified OHE may be inappropriate for undergraduate students, who have a relatively high ability to learn and acquire professional knowledge. Oral health education regarding periodontal health (OHE-PH), originally designed for dental students, contains information on the prevention and maintenance of periodontal diseases [20, 21]; OHE-PH differs from other popularized science education, providing a more comprehensive periodontal health knowledge system. However, it is not clear that the effects of OHE-PH on non-dental undergraduates who are prime targets of such education.

We may be confronted with another problem, even if OHE-PH is effective for undergraduates. The Chinese university student population in 2019 was very large: 30.32 million [22]. Many colleges and universities do not offer a major in dentistry; thus, it is difficult to develop face-to-face oral health education, let alone OHE-PH. E-learning courses seem to be an alternative way to educate these students.

E-learning refers to a learning and teaching method that uses electronic technology [23, 24]; it is also termed web-based learning or training, online learning or education, Internet-based learning, multimedia learning, technology-enhanced learning, virtual learning, and computer-assisted, computer-aided, or computer-based instruction [25]. Compared with traditional courses, e-learning has the advantage of flexibility in learning [26]. Students can use it on demand without being limited in terms of time, place, pace and scale [27].

E-learning has been widely applied in medical education [26, 28–30] and is as effective as other learning methods [28, 31]. E-learning already plays a role in dental education, such as oral radiology [27, 32] and orthodontics education [33]. These e-learning courses are part of compulsory curriculum oriented towards dental students only. In addition, e-learning has been adopted in oral health promotion and has improved oral health knowledge among children [34]. However, the effect of e-learning on OHE-PH for adults, especially non-dental students, is not clear.

In this study, we implemented a traditional OHE-PH course and an e-learning OHE-PH course for non-dental students to examine the effect of OHE-PH on non-dental students and the effect of an e-learning course on OHE-PH.

## Methods

### Design

A quasi-experimental study with a pre-test and a post-test group was performed for this research.

### Participants

The inclusion criterion was being a third-year undergraduate dental student (the first year in dental education) or second through fourth-year non-dental undergraduate enrolling in the optional course “Oral Prophylaxis and Hygiene” at Sichuan University. The exclusion criterion was being a student who did not consent to participate in the survey.

Comparing the means of two independent samples, the calculation n = 2[(z_1−α/2_ + z_1−β_) σ/(μ_t_ − μ_c_)]^2^ was applied [35]. A pre-test regarding the participants’ knowledge of periodontal disease before and after the course was administered to determine σ, μ_t_ and μ_c_. The samples were estimated to number more than 50 in each group.

### Ethics approval and consent to participate

This study was approved by the Institutional Review Board of the Ethics Committee of the West China Hospital of Stomatology, Sichuan University (WCHSIRB-D-2018-092). All the participating students signed an informed consent form. All methods were performed in accordance with the relevant guidelines and regulations.

### Intervention and instruments

The traditional periodontal health education was arranged as a 90-min course. The content was designed based on the textbook “Preventive Dentistry” [20, 21]. Both the dental students and non-dental students took the traditional course in 2019. The e-learning course was based on the online components of the “Preventive Dentistry” textbook [36]. The non-dental students who enrolled in 2020 received a total of 45-min of online course time and a 45-min online question and answer session via Tencent Group. When explaining the aetiology of periodontal diseases, the corresponding prevention (plaque control) and clinical treatment were also introduced. Periodontitis-associated systemic diseases were also explained. To enhance understanding and attention, modifications were made to the course content by including some animation and videos instead of text explanations.

All students received the pre-course survey before the lecture. To avoid the students exchanging information as much as possible, before the survey, we informed students that they were required to complete the survey independently and that the results were irrelevant to their final score in the course. The first section of the survey included an 11-question knowledge test on periodontal diseases using a 5-point Likert-type scale ranging from 1 (strongly disagree) to 5 (strongly agree). The 11 questions were sorted into 5 items (self-evaluation of their knowledge of periodontal diseases; aetiology and risk factors for periodontal diseases; periodontal health and systematic health; possible outcomes of tooth in elderly individuals; treatment of periodontal diseases). The second part included 4 items on sources of knowledge on periodontal diseases, the students’ gingival bleeding history and self-reported behaviours regarding gingival bleeding and oral care.

A post-course survey including the same 11-question knowledge test and 2 questions on self-reported behaviours related to gingival bleeding and oral care was administered immediately after the course. For students taking the e-learning course, the post-course survey also included 3 items regarding attitudes towards the e-learning course. The variables included the different majors of students (dental students and non-dental students) and the OHE-PH teaching method (the traditional course and the e-learning course). All outcomes were self-reported through the questionnaire. The survey was created and managed by a universal questionnaire designer (www.wjx.com).

### Data collection and analysis

The Cronbach’s alpha coefficient was analysed using SPSS 22.0 (IBM Corp. New York, NY, USA). Six experts (1 professor, 3 associate professors and 2 lecturers) assessed the content validity [37], clarity, and conciseness of the instrument. The data are presented as percentages, means and standard deviations (SD). The Mann–Whitney U test, the chi-square test and Fisher’s exact test were used for statistical analysis in SPSS 22.0. *P* < 0.05 was regarded as a statistically significant difference.

## Results

Ultimately, the pre-course survey was taken by 217 third-year undergraduate dental students (86 males, 131 females; 21.32 ± 1.04 years old) and a total of 203 non-dental students (90 males, 113 females; 20.57 ± 1.31 years old). For the post-course survey, 1 non-dental student was excluded for improper answers. The non-dental students were in the 2nd–4th years of their program. The majors of the non-dental students are shown in Additional file 1.

**Fig. 1:**
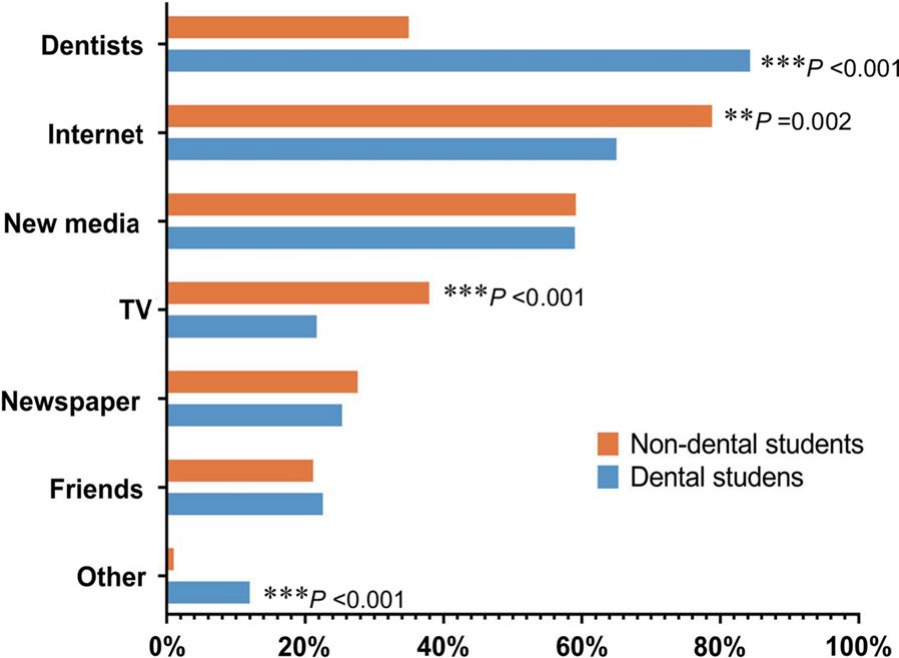
Sources of knowledge on periodontal diseases for dental and non-dental students (Chi-square test; ***P < 0.001; **P < 0.01)

Reliability analysis of the items regarding knowledge of periodontal diseases showed that the Cronbach’s alpha coefficients were 0.781 for the pre-course survey and 0.711 for the post-course survey. A content validity index (CVI) was calculated for the questionnaire items. The item-level CVI (I-CVI) and the scale-level CVI (S-CVI) were 1.

We tested 11 questions (5 items) regarding the aetiology, outcome and treatment of periodontal diseases (Table 1). Encouragingly, dental (*P* < 0.001, except item 3 *P* = 0.003) and non-dental students (*P* < 0.001) showed significant improvements after completing the course. Moreover, non-dental students taking the traditional course achieved scores similar to (Items 2–4) or surpassing (item 1, *P* < 0.001) the level of dental students before the course. The e-learning course had unexpected effects. The students improved their knowledge of periodontal health through the e-learning course. The baselines of the scores of students attending the e-learning course and the traditional course remained consistent (*P* > 0.05). After the course, the score of students taking the e-learning course was higher than that of students taking the traditional course for items 2, 3 and 4 (*P* < 0.05). There were no significant differences in other items.

**Table 1:** Dental and non-dental students’ knowledge of periodontal diseases before and after a traditional or e-learning course

Items	Dental students			Non-dental students					
	Traditional course			Traditional course			E-learning course		
	Before (n = 217)	After (n = 217)	*P*^a^	Before (n = 134)	After (n = 134)	*P*^b^	Before (n = 69)	After (n = 68)	*P*^c^
Self-evaluation about the knowledge of periodontal diseases	3.98 ± 0.51	4.87 ± 0.34	< *0.001*	2.82 ± 0.87	4.27 ± 0.73	< *0.001*	2.93 ± 0.96	4.19 ± 0.43	< *0.001*
*P*^d^							0.429	0.252	
*P*^e^					< *0.001*				
Aetiology and risk factors for periodontal diseases	11.99 ± 0.96	12.82 ± 0.42	< *0.001*	10.79 ± 1.27	12.09 ± 1.17	< *0.001*	10.88 ± 1.16	12.38 ± 0.88	< *0.001*
*P*^d^							0.618	*0.036*	
*P*^e^					0.235				
Periodontal health and systematic health	13.63 ± 1.18	13.93 ± 1.20	*0.003*	11.71 ± 1.39	13.52 ± 1.65	< *0.001*	11.91 ± 1.67	14.04 ± 1.37	< *0.001*
*P*^d^							0.413	*0.012*	
*P*^e^					1				
Possible outcomes of tooth in elderly individuals	3.71 ± 0.98	4.37 ± 0.92	< *0.001*	3.01 ± 0.99	3.85 ± 1.11	< *0.001*	3.03 ± 0.97	4.21 ± 0.86	< *0.001*
*P*^d^							0.750	*0.028*	
*P*^e^					0.077				
Treatment of periodontal diseases (Scaling)	12.48 ± 1.53	13.47 ± 1.47	< *0.001*	11.19 ± 1.99	12.78 ± 1.90	< *0.001*	11.29 ± 2.16	13.06 ± 1.80	< *0.001*
*P*^d^							0.529	0.232	
*P*^e^					0.075				

Mann–Whitney U test; *P* < 0.05 values were significant

^a^Comparison of dental students before and after a traditional course

^b^Comparison of non-dental students before and after a traditional course

^c^Comparison of non-dental students before and after an e-traditional course

^d^Comparison of non-dental students before/after a traditional course and non-dental students before/after an e-learning course

^e^Comparison between non-dental students after a traditional course and dental students before a traditional course

Later, we surveyed the sources of knowledge about periodontal diseases (Fig. 1). The top three sources of dental students were ‘dentists’, ‘the Internet’, and ‘new media (WeChat, Weibo etc.)’. For non-dental students, the top three sources were ‘the Internet’, ‘new media (WeChat, Weibo etc. on one’s cell phone)’ and ‘TV’. Dental and non-dental students had obvious differences in their responses regarding ‘dentists’ (*P* < 0.001), ‘the Internet’ (*P* = 0.002) and ‘TV’ (*P* < 0.001). The most important source of information for dental students was ‘dentists’ (84.33%), while 34.98% of non-dental students acquired oral periodontal health knowledge from ‘dentists’. Conversely, most non-dental students obtained knowledge from the Internet (78.82%).

Gingival bleeding is a common symptom of periodontal diseases, as it is an indication that inflammation is occurring at the gingiva [38]. We surveyed the students’ bleeding symptoms when brushing their teeth over the most previous month (Additional file 2). We found that 15.76% of non-dental students frequently suffered from gingival bleeding, while 6.45% of dental students had experienced gingival bleeding frequently in the last month (*P* = 0.001). Non-dental students suffered from more frequent gingival bleeding in the last month than dental students had (*P* = 0.001).

**Fig. 2:**
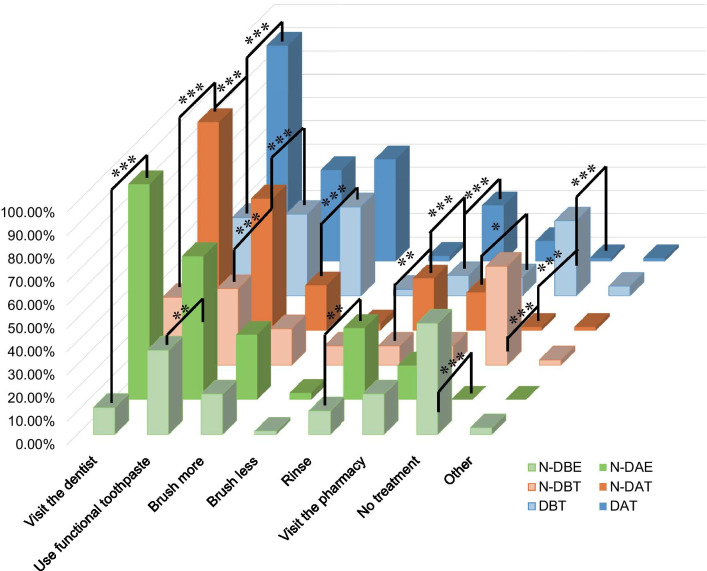
Treatment for gingival bleeding among dental and non-dental students before and after a traditional or e-learning course. *N-DBE* non-dental students before the e-learning course, *N-DAE* non-dental students after the e-learning course, *N-DBT* non-dental students before the traditional course, *N-DAT* non-dental students after the traditional course, *DBT* dental students before the traditional course, *DAT* dental students after the traditional course. Chi-square test when n > 5; Fisher’s exact test when n <  =5; ***P < 0.001; *P < 0.05)

The treatment for gingival bleeding may influence the final outcome: gingivitis or periodontitis. Proper treatment will relieve gingivitis, while some uncontrolled gingivitis may progress to irreversible periodontitis, which can ultimately lead to tooth loss [39, 40]. Figure 2 shows the possible treatments for students suffering from gingival bleeding. After the traditional course, it was encouraging to find that only few students refused to be treated for gingival bleeding. The first treatment choice of dental and non-dental students was visiting a dentist. Brushing more was the second choice of dental students, while using a functional toothpaste was the second choice for non-dental students. More dental students chose ‘visit the dentist’ and ‘rinse’ after the traditional course than had chosen such treatment options before the course (*P* < 0.001). After the course, there was a significant increase in the number of non-dental students who chose to visit the dentist (traditional course: *P* < 0.001; e-learning course: *P* < 0.001), use functional toothpaste (traditional course: *P* < 0.001; e-learning course: *P* < 0.001) and rinse (traditional course: *P* = 0.001; e-learning course: *P* < 0.001). More non-dental students chose to visit the dentist (*P* < 0.001), use functional toothpaste (*P* < 0.001), rinse (*P* < 0.001) and go to the pharmacy (*P* = 0.015) after taking the traditional course than had dental students before the course. In addition, the e-learning course group was similar to the traditional course group for all treatments except visiting the dentist because the traditional course group was already more willing to go to the dentist for cases of gingival bleeding before the course (*P* = 0.005).

**Fig. 3:**
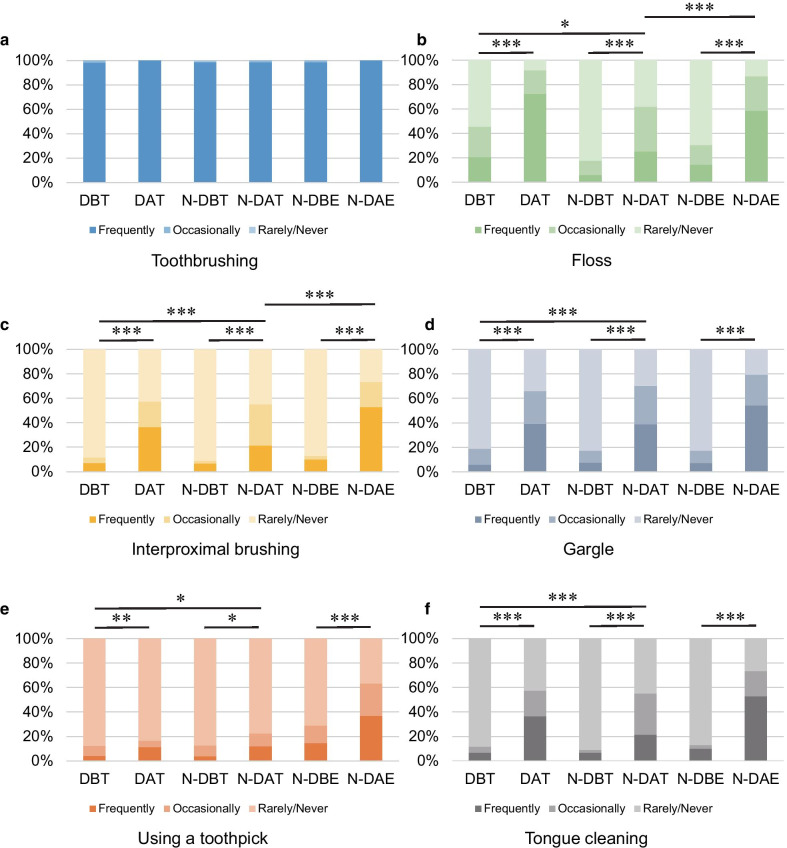
The frequency of use of oral care methods by dental and non-dental students before and after a traditional or e-learning course. **a** Toothbrushing, **b** flossing, **c** interproximal brushing, **d** gargling, **e** using a toothpick, **f** tongue cleaning. *N-DBE* non-dental students before the e-learning course, *N-DAE* non-dental students after the e-learning course, *N-DBT* non-dental students before the traditional course, *N-DAT* non-dental students after the traditional course, *DBT* dental students before the traditional course, *DAT* dental students after the traditional course. Chi-square test when n > 5; Fisher’s exact test when n≦5; ***P < 0.001; **P < 0.01; *P < 0.05)

Finally, the self-reported behaviours for oral care (toothbrushing, gargle, floss, interproximal brushing, using a toothpick and tongue cleaning) were surveyed (Fig. 3). The results showed that almost 100% of students acknowledged the value of and used toothbrushes before and after the course. After the course, dental and non-dental students increased their frequency of flossing, using interproximal brushes, gargling and cleaning their tongues (*P* < 0.001). In addition, after taking the traditional course, non-dental students were more likely than dental students before the course to floss (*P* < 0.05), use an interproximal brush (*P* < 0.001), gargle (*P* < 0.001) and clean their tongue (*P* < 0.001). However, more dental students (*P* < 0.01) and non-dental students (traditional course: *P* < 0.05; e-learning course: *P* < 0.001) reported using toothpicks after the course than had done so the course. The e-learning course group tended to use floss and interproximal brushes more frequently than those who received the traditional course (*P* < 0.001). A comparison between the effects of the traditional and e-learning courses on toothpick use and tongue cleaning is not shown, as the baseline (before class) was different.

The attitudes of the e-learning course group towards the e-learning course compared to towards traditional courses were surveyed. The results showed that 75.00% of students preferred the e-learning course and 98.53% of students thought e-learning was helpful for them. Repeatability, convenience and novelty were the top 3 advantages of e-learning, while the inadequate of learning atmosphere and interaction among students and between students and teachers were the top 3 disadvantages.

## Discussion

For dental students at Sichuan University, the prevention of periodontal diseases was part of a compulsory course, “Preventive Dentistry” [20, 21]. The course was designed based on the aetiology of periodontal diseases. Given their similar primary-, junior- and high-school educational backgrounds, dental and non-dental undergraduates in a Chinese university have similar general knowledge and conceptions [17]. It is possible for non-dental students to attend a similar course to one that dental students attend. OHE-PH includes the aetiology, symptoms, and treatment of periodontal diseases and methods of tooth cleaning, which contribute to a more thorough understanding than a simplified education would. During the 2019–2020 school year, non-dental students took a traditional OHE-PH course or an e-learning OHE-PH course.

We tested the students’ prior knowledge about periodontal diseases. After the course, all the students had improved knowledge. After the course, non-dental students’ scores met or exceeded the baseline scores of dental students. The course ensured an acceptable improvement among non-dental students, suggesting that a well-designed course could be offered to both dental and non-dental university students. The non-dental students who took the e-learning course improved their periodontal health knowledge, and it was surprising that students taking the e-learning course performed better than those taking the traditional course in some respects.

Figure 2 shows the possible treatments offered for gingival bleeding. Brushing is more effective when gingivitis first begins [41]. Using functional toothpaste has an auxiliary function to tooth brushing. However, visiting the dentist is also important when dental calculus forms. The largest behavioural difference between dental and non-dental students related to brushing. Non-dental students tended to brush less and avoid brushing more than dental students, partially reflecting the fact that the Chinese public lacks an awareness about the relationship between brushing and gingivitis [42]. Additionally, choosing not to pursue treatment was a common choice among both dental and non-dental students before the course. All the students improved their self-reported behaviours for the treatment of gingival bleeding after the traditional or e-learning course. Visiting the dentist became the top choice for both groups. Non-dental students still did not grasp the idea that brushing more would alleviate gingival bleeding; thus, this content area could be improved in the future.

We also surveyed the frequency of use of oral care methods. All the students reported positive tooth brushing behaviours (Fig. 3). However, approximately 80% of non-dental students had rarely/never used floss or gargled before the course. This suggests that oral care education for Chinese students is far from sufficient. Fortunately, all the students dramatically improved their oral care habits after the course. Non-dental students were still less likely to use floss, indicating that more emphasis should be placed on the importance of this behaviour. The frequency of toothpick use increased among non-dental students after both the traditional and e-learning course, which indicated that OHE-PH on reducing the use of toothpicks should be strengthened to avoid periodontal injury [17]. Interestingly, non-dental students attending the e-learning course had a more positive attitude towards using floss and interproximal brushes than those taking the traditional course, demonstrating that the e-learning course can improve oral self-reported behaviours for oral care.

Regarding knowledge sources, dentists were undoubtedly the most common source of information for dental students, who had taken some related courses before. Non-dental students preferred to obtain knowledge from the Internet, new media (WeChat, Weibo, etc.) and TV rather than from other sources. The Internet, especially oral health-related websites, has helped improve attitudes and knowledge among kindergarten/elementary school teachers and their students [43]. WeChat, a public Chinese social media platform, has gradually become used for university education, and 45.4% of its users are between 18 and 25 years old [44, 45]. However, the information spread through the Internet and new media may carry many inaccuracies [46]. Ultimately, it is feasible to offer e-learning courses to non-dental students through the Internet and new media (WeChat, Weibo, etc.).

The results suggested that e-learning courses may be a promising development in OHE. As they are repeatable and convenient, e-learning courses might improve learning motivation, ensuring that students study what they like or what they want to learn. Even though the e-learning course was inadequate in terms of interaction [23], the option for simultaneous online communication partly compensated for this deficiency. Additionally, e-learning courses about OHE may be offered to teachers and school doctors, especially those working in kindergartens and primary schools, so that they can provide OHE for students instead of dentists having to come in to do so [15]. E-learning courses play an important role in times of crises such as the SARS, H1N1v and Ebola outbreaks [24]. Many universities have used e-learning for undergraduates during the COVID-19 pandemic [47, 48]. E-learning courses might become more popular when face-to-face education is not an option.

E-learning is beneficial for populations in which good health and well-being and quality education are targeted, especially in developing countries [49]. In China, there are on 637,000 dentists [23], and they are responsible a very large population of 1.4 billion people [24, 25]. Thus, the density of dentists in China is far below that in Japan (7.95 dentists per 10,000 population) or Germany (8.52 dentists per 10,000 population) [25]. The rate is even below the standard set by the WHO, which is 1:5000 [26]. It is very insufficient to depend only on Chinese dentists to provide face-to-face OHE. E-learning may enable the provision of OHE for extended populations nationwide as an auxiliary or even main teaching method to increase knowledge about oral health if such e-learning courses are made available to the public.

There are significant merits to using e-learning courses on OHE-PH to promote OHE. However, there are challenges in extending e-learning courses. Institutes such as universities and hospitals may encounter increased IT infrastructure costs to support the development of e-learning. Additionally, educators need to routinely update their courses as dentistry knowledge advances [23].

### Limitations

The study had several limitations. First, it was a quasi-experimental study, as the number of non-dental students in 2020 was much lower than that in 2019, and it was difficult to ensure the representativeness of samples, which led to differences in the baseline of some items. Second, the post-course survey was conducted within a short period. Long-term changes in students’ knowledge and attitudes are unknown. Additionally, some interfering factors (the participants’ sociodemographic characteristics) were not considered in this study, which might cause bias. Finally, it was difficult to ensure the consistency of the learning environment for non-dental students attending online courses, especially when students were at home during the COVID-19 pandemic.

## Conclusions

OHE-PH via a traditional course was effective for both dental and non-dental students. OHE-PH via an e-leaning course was not only an acceptable and feasible method for educating non-dental students but was as effective for non-dental students as the traditional course and even better in some areas. Thus, the e-learning course on OHE-PH might be a feasible method for helping undergraduates improve their periodontal health knowledge and attitudes.

## Supplementary Information

**Additional file 1:** Supplementary Figure 1. The distribution of majors of non-dental students.

**Additional file 2:** Supplementary Figure 2. Bleeding when brushing among dental and non-dental students in the last month.

## Data Availability

The datasets supporting the conclusions of this article are included within the article and its additional files.
