# Supplementary material for: The effects of oral health education regarding periodontal health on non-dental undergraduates in southwestern China—exploring the feasibility of an e-learning course for oral health promotion
Source: BMC Oral Health. 2021 Mar 16;21:119. doi: 10.1186/s12903-021-01476-5 (PMC7962077; doi:10.1186/s12903-021-01476-5)
Supplement: Supplementary file 2 — Additional file 2: Supplementary Figure 2. Bleeding when brushing among dental and non-dental students in the last month. [file 12903_2021_1476_MOESM2_ESM.pdf]

The effects of oral health education regarding periodontal health on non-dental undergraduates in southwestern China—exploring the feasibility of an e-learning course for oral health promotion

Zhiwu Wu[1#], Mingming Li[1#], Fangzhi Zhu[1], Lei Lei[1], Ran Cheng [1*], Tao Hu[1*]

[1]Department of Preventive Dentistry, State Key Laboratory of Oral Diseases, West China Hospital of Stomatology, Sichuan University, Chengdu, Sichuan, China;

# The authors contribute equally to the work

* Corresponding authors:

Ran Cheng

Department of Preventive Dentistry, State Key Laboratory of Oral Diseases, West China Hospital of Stomatology, Sichuan University, Chengdu, Sichuan, China; Tel: 86-28-85503486; E-mail: chengran@scu.edu.cn

and

Tao Hu

Department of Preventive Dentistry, State Key Laboratory of Oral Diseases, West China Hospital of Stomatology, Sichuan University, Chengdu, Sichuan, China; Tel: 86-28-85503486; E-mail: hutao@scu.edu.cn

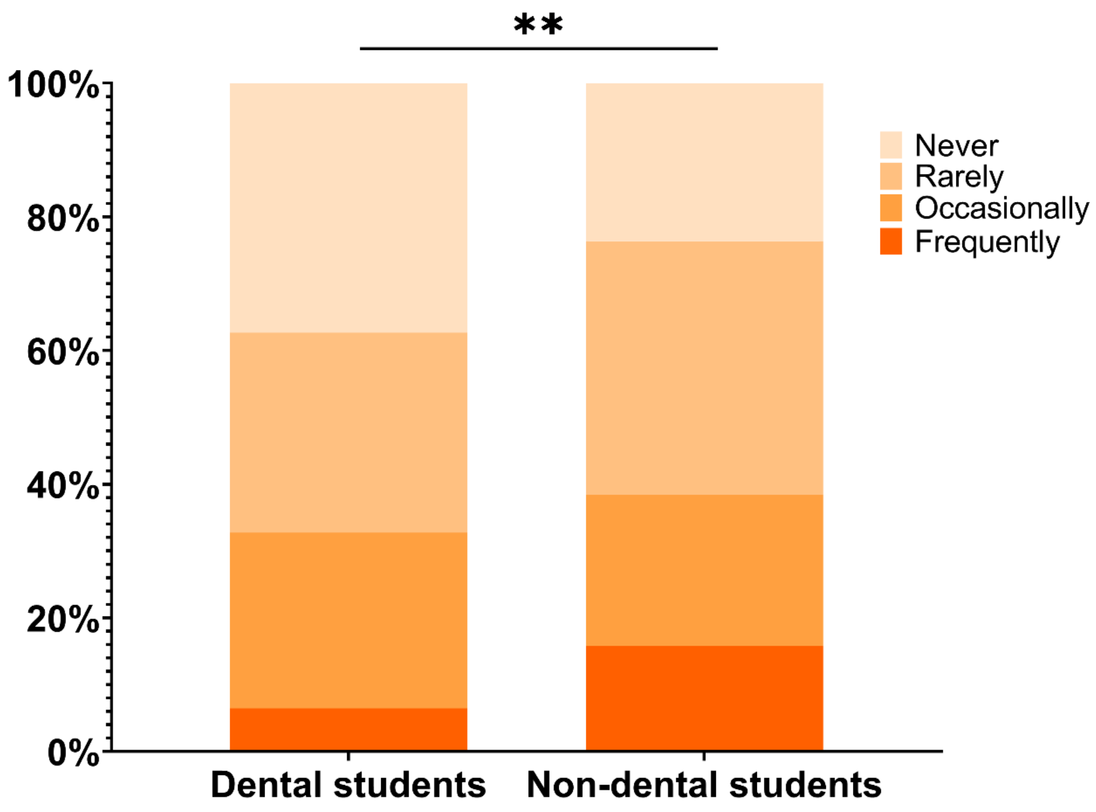

Supplementary Figure 2. Bleeding when brushing among dental and non-dental students in the last month (Chi-square test; ** *P* <0.01).
